# Mental health peer support relationship: a realist informed qualitative meta synthesis

**DOI:** 10.1136/bmjopen-2025-105211

**Published:** 2025-12-30

**Authors:** Corinna Hackmann, Amanda Green, Ruby Pease, Izobel Clegg, Melanie Handley

**Affiliations:** 1 Norfolk and Suffolk Foundation Trust, Norwich, UK; 2 University of East Anglia, Norwich, UK; 3 King’s College London, London, UK; 4 University of Hertfordshire, Hatfield, UK

**Keywords:** MENTAL HEALTH, Protocols & guidelines, Health Workforce, Empathy

## Abstract

**Abstract:**

**Objective:**

To understand how key relational factors lead to observed outcomes in mental health peer support.

**Design:**

This realist-informed qualitative review synthesised findings from 18 studies to develop programme theory relating to interpersonal contexts, linked outcomes and underlying mechanisms of change of mental health peer support.

**Data sources:**

Four databases were searched: PsycINFO, Embase, Medline, CINAHL.

**Eligibility criteria for selecting studies:**

All studies were evaluated for relevance and rigour for development of programme theory.

**Data extraction and synthesis:**

Qualitative data were extracted from 20 studies. A realist-informed synthesis identified repeating themes with context-mechanism-outcome configurations.

**Results:**

This identified five key contextual factors that together form the APPEAR framework (Accepting, Personalised Practice, Empowering, Available and Reciprocal). These contextual factors were found to interact to create the conditions for improved personal recovery outcomes in the domains of (1) self-acceptance, (2) confidence, (3) hopefulness, (4) self-expression, (5) relationships and (6) knowledge and skills.

**Conclusions:**

The APPEAR framework offers an operational foundation for understanding interpersonal mental health peer support interventions.

**Strengths and limitations of this study**

- This literature synthesis is a novel methodological approach that integrates thematic synthesis with realist research methods.
- The approach enabled development of a theoretical framework based on the relationships between contexts and causal mechanisms that underlie the impacts of mental health peer support.
- Stakeholders were consulted during analysis to ensure that theory development was meaningful and useful, but the resulting framework would benefit from systematic empirical testing and refinement.

## Introduction

 Peer support workers are now a common and established feature of mental health services globally.^1 2^ The World Health Organization (WHO) has endorsed the importance of peer support to facilitate person-centred community mental healthcare delivery (WHO 2012, guidance on Community MH services). Mental health peer support involves individuals with relevant lived experience offering structured support to others with similar challenges. Formal peer support has been linked with a range of outcomes including improved mental health,^1^,^3^ reduced social isolation,^2 4^ increased hope^5 6^ and increased feelings of agency and empowerment.^5 6^ These outcomes align with the construct of personal recovery, which has been defined as building ‘satisfying, hopeful and contributing lives, even with the limitations caused by illness’.^7^

The role of the peer relationship has been described as critical to the outcomes achieved by such interventions. Repper *et al* characterise peer support as ‘offering and receiving help, based on shared understanding, respect and mutual empowerment between people in similar situations’.^8^ Other authors have highlighted the importance of mutual understanding,^3 5^ trust^4 9^ and reciprocity.^1 5^ Qualitative research best captures the nuance of people’s perspectives and experience. Qualitative studies have described both key interpersonal dynamics and nuanced recovery outcomes resulting from formalised peer support. What is not currently known is *how* different interpersonal dynamics lead to the observed outcomes. Understanding what it is about how relationships between peer support workers and those they work with in mental health services develop and are sustained can inform when and with whom peer support interventions are offered. Given the increasing use of peer support workers within existing mental health service delivery, a realist informed literature synthesis was warranted.

This study aimed to address a gap in the current understanding of how peer support interventions achieve the observed outcomes by systematically synthesising qualitative literature describing the peer support relationship and its impacts. This involved the integration of thematic synthesis^10^ to identify prominent themes, with realist review methods^11 12^ to produce causal explanations that set out the relationships between contexts and mechanisms that underlie outcomes.

### Objectives

Identify and synthesise qualitative literature on mental health peer support workers and their role in mental health services.

Build and refine the theoretical framework with peer support workers from the evidence and in consultation with stakeholders.

## Method

A synthesis of qualitative data was undertaken to build evidence-based theories of peer support workers and their role in mental health services. This involved initially synthesising the data to identify repeating themes relating to the descriptions of the peer relationship.^10^ A realist-informed approach was used to identify repeating patterns between these interpersonal contexts (such as mutual understanding and trust) and outcomes observed (such as decreased social isolation and increased feelings of agency and control) and develop an evidence-based theoretical understanding of how and why these observed contexts and outcomes co-occur.^11 12^ This will be used to develop practical guidance for peer support workers and wider mental healthcare delivery as well as for people who are accessing and using peer support services.

### Search strategy and selection criteria

Four databases (PsycINFO, Embase, Medline and CINAHL) were searched from January 2000 to June 2023 and updated in May 2024. The year 2000 date restriction reflects the development of peer support workers in services to explicitly support personal recovery (see online supplemental appendix 1 S1, for full search strategy).

Inclusion criteria comprised primary research with a formal qualitative component exploring the experience of formalised face-to-face individual peer support interventions from the perspective of peer support workers or service users. Included studies explored peer support interventions delivered to adult mental health service users accessing statutory or private mental health services or charitable organisations. To ensure theory development retained relevance to the topic of mental health, we excluded peer support interventions for dementia, developmental disorders, substance use, physical conditions, injury, learning disabilities, carers and any participants under 18 years old. We also excluded studies which explored peer interventions that were educational or learning programmes, or were aimed solely to support grief, physical health conditions, parenting or veterans returning to civilian life. We excluded non-formalised approaches (ie, naturally occurring), peer support that was not delivered face-to-face (ie, online or over the phone) and group delivery. We searched the first 10 pages of Google Scholar and reviewed the references of included papers.

Two authors completed a title and abstract screening (PR and IC). Ten per cent of articles were reviewed together to ensure inter-rater reliability. All uncertain citations were included for full-text review. All articles for full-text screening were reviewed by both authors. Uncertainties were resolved with a third author (CH).

### Data extraction

**Figure 1:**
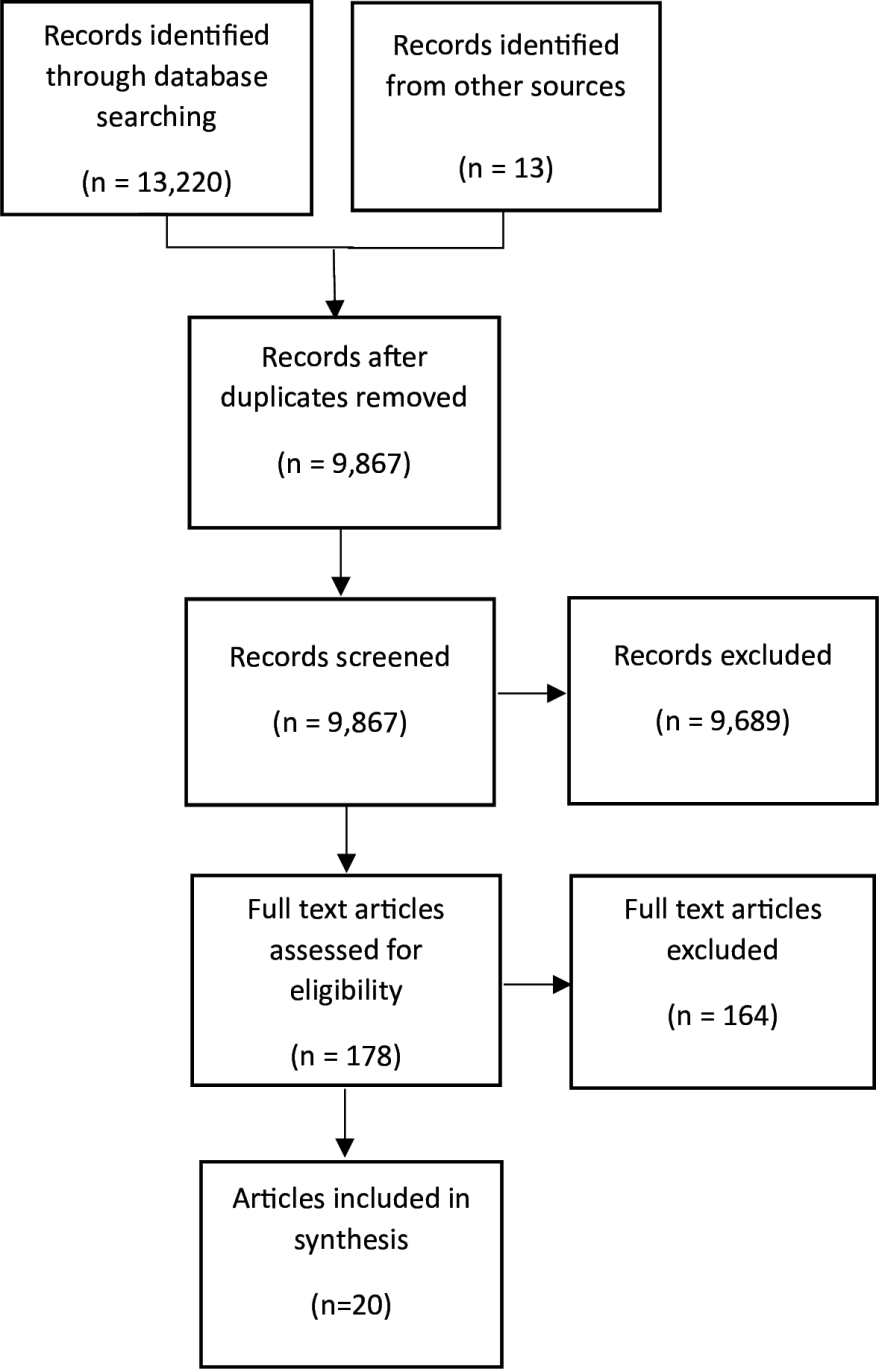
PRISMA flow diagram of included literature. PRISMA, Preferred Reporting Items for Systematic reviews and Meta-Analyses.

Three authors extracted data (RP, IC and CH). Independent initial data extraction was compared for three random papers to verify reliability of data extraction; any disagreements were discussed and resolved. Demographic, service setting and methodological data were extracted into a prepiloted table (see online supplemental table 1 S2, Study and participant demographics). Rigour was not directly assessed; instead, inclusion was based on relevance to understanding the peer relationship and developing programme theory.^13^ See figure 1 for preferred reporting items for systematic reviews and meta-analyses flow diagram of included literature.

### Coding and synthesis

This review sought to first thematically synthesise qualitative data on the peer relationship^10^ to identify the key relational factors and second, use realist-informed methods^11 12^ to identify the outcomes related to these relational contextual factors and the potential mechanisms underlying this. This is reported in line with enhancing transparency in reporting the synthesis of qualitative research ENTREQ reporting standards for qualitative synthesis ^14^(see online supplemental table 2 S3, ENTREQ Checklist) and Realist and Meta-narrative Evidence Syntheses: Evolving Standards RAMESES publication standards for realist methods ^15^(see online supplemental table 3 S4, RAMESES checklist)

**Figure 2:**
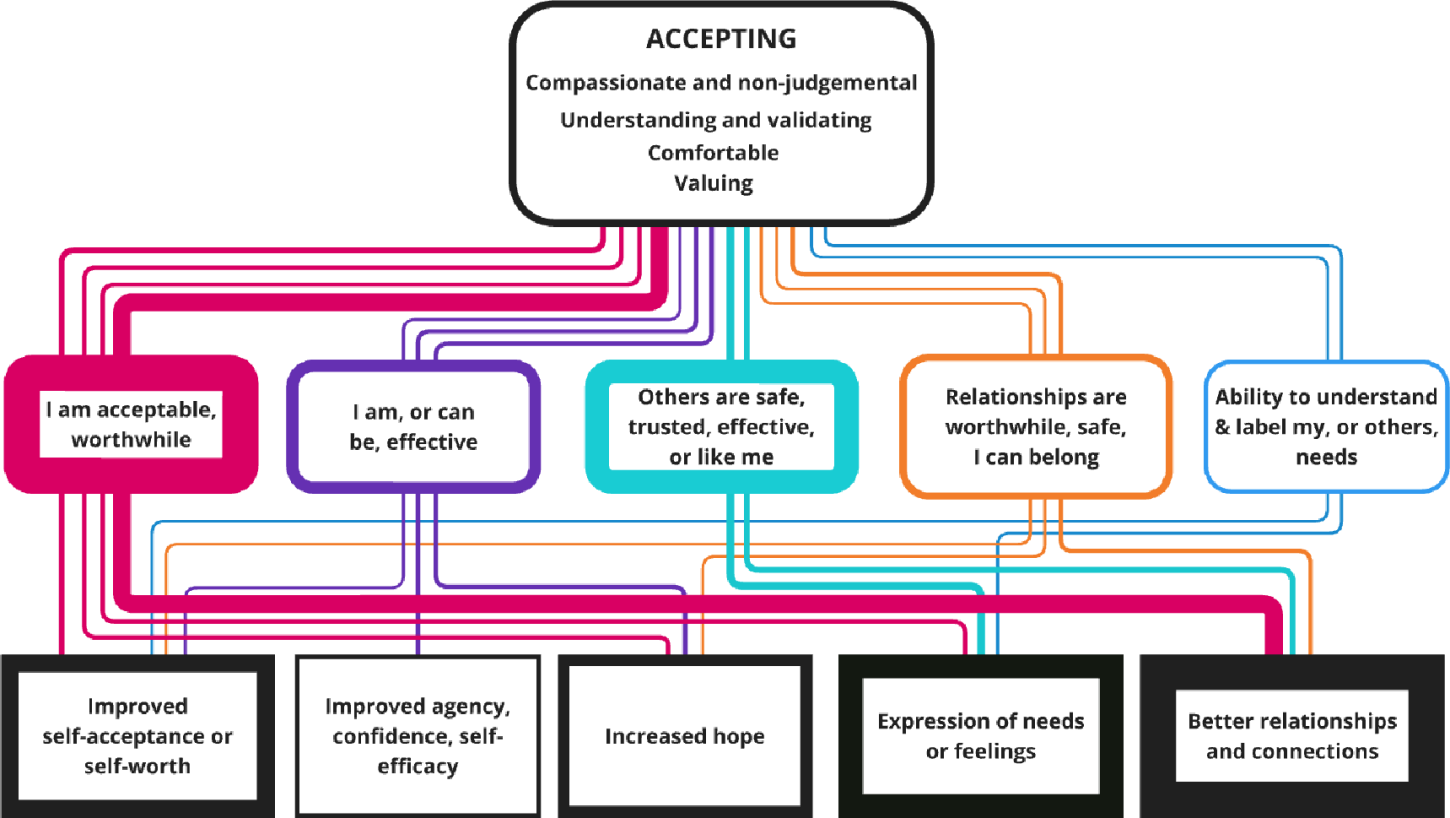
Nested CMOCs for the accepting interpersonal context. Represents the interpersonal factors included in the accepting context. The coloured soft rectangles represent the mechanisms, and hard rectangles are the outcomes. The thickness of borders and lines represents the number of times mechanisms and outcomes were coded or linked. CMOC, context–mechanism–outcome configurations.

Qualitative data on the experiences and perspectives of service users and peer support workers were synthesised to capture key dimensions of the relationship. We initially coded, line-by-line, descriptions and observations of the nature of the relationship between peer support workers and service users. This was categorised into ‘descriptive’ themes, which were close to the qualitative data.^10 16^ These were iteratively developed into ‘analytical themes’ of pivotal relational contexts to represent superordinate or analytical constructs.^10^ Repeating patterns of interpersonal contexts being described with or ascribed to outcomes (defined as observable impacts aligned with well-being, mental health or personal recovery) were identified along with potential underlying mechanisms. These formed our initial context–mechanism–outcome configurations (CMOCs) and coding framework. We coded both data that separately identified interpersonal contexts and outcomes of the peer relationship and nested CMOCs (ie, where interpersonal contexts, outcomes and mechanisms were identified together in the data). This was an iterative process of refining the descriptive and analytical themes of relational contexts, and the patterns of CMOCs, as more data were added (see online supplemental table 4 S5, for the codes and themes for each article and online supplemental table 5 S6, for data examples from included articles with CMOC coding). Competing explanations were considered through constant iterative comparison between data sources and the evolving themes and related CMOCs. Alternative interpretations were discussed within the research team and assessed for relevance, explanatory power and alignment with the articles in the review. The nested CMOCs were used to develop infographics for each explanatory interpersonal context (see figures2^6^).

**Figure 3:**
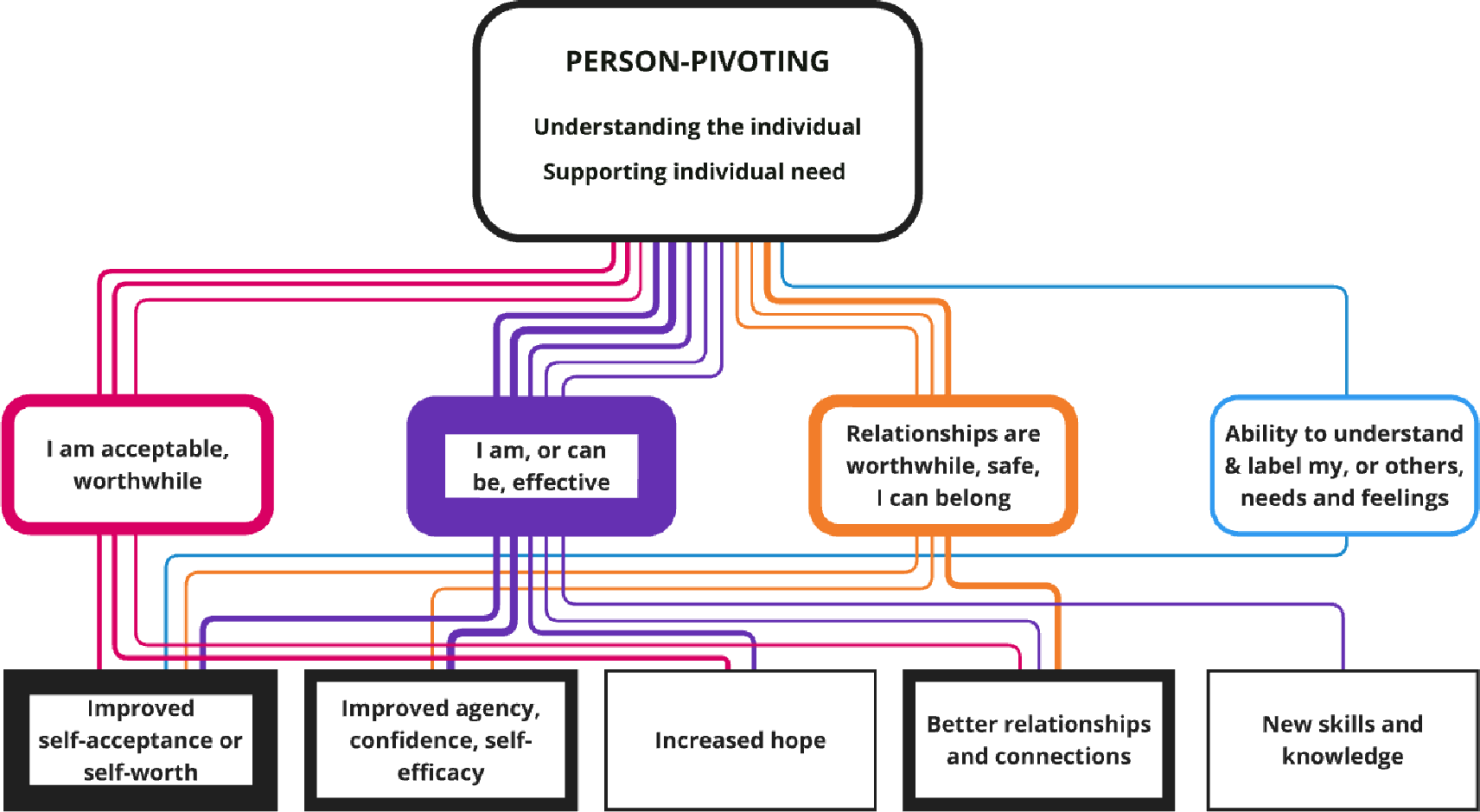
Nested CMOCs for the Personalised Practice interpersonal context. Represents the interpersonal factors included in the Personalised Practice context. The coloured soft rectangles represent the mechanisms, and hard rectangles are the outcomes. The thickness of borders and lines represents the number of times mechanisms and outcomes were coded or linked. CMOCs, context–mechanism–outcome configurations.

**Figure 4:**
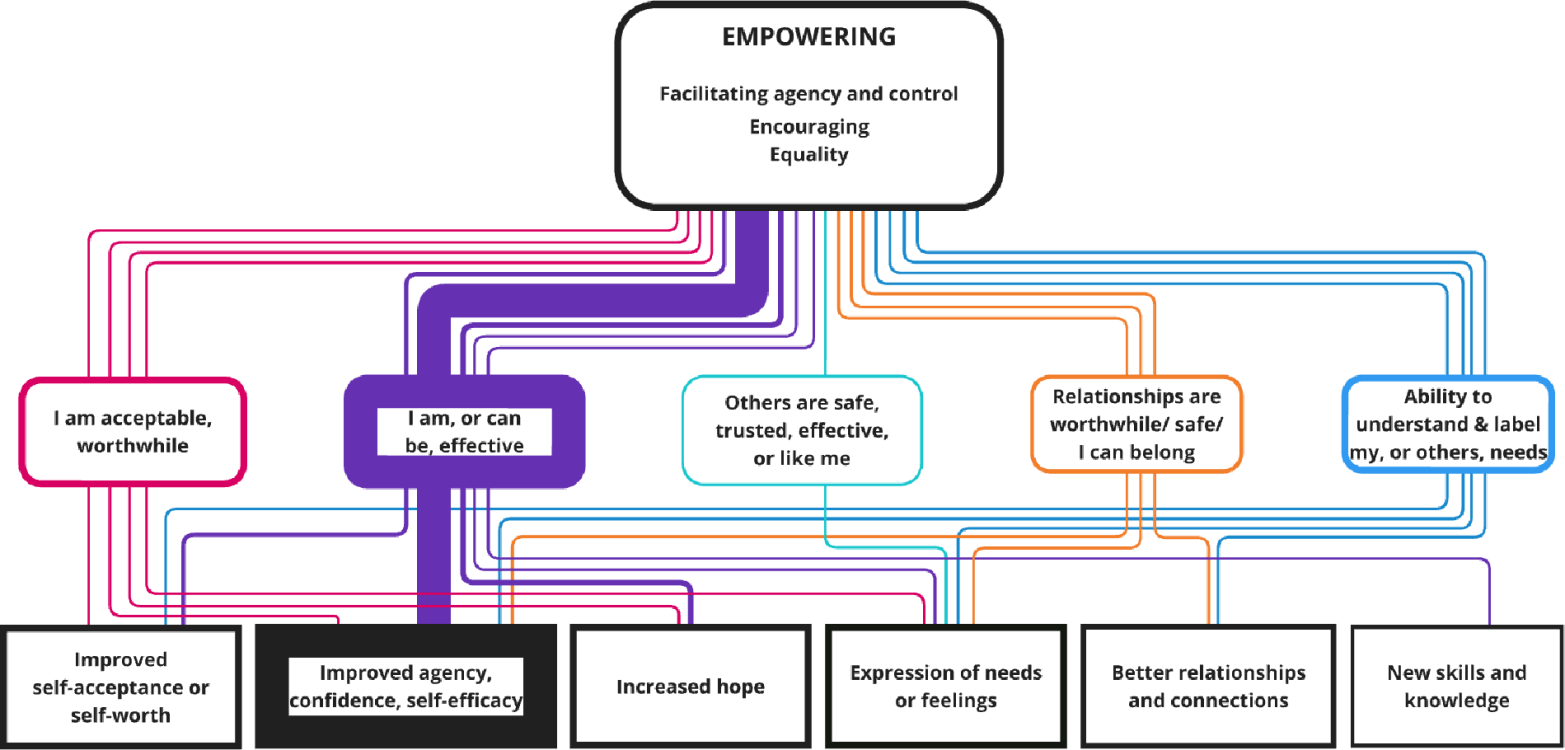
Nested CMOCs for the Empowering interpersonal context. Represents the interpersonal factors included in the Empowering context. The coloured soft rectangles represent the mechanisms, and hard rectangles are the outcomes. The thickness of borders and lines represents the number of times mechanisms and outcomes were coded or linked. CMOCs, context–mechanism–outcome configurations.

**Figure 5:**
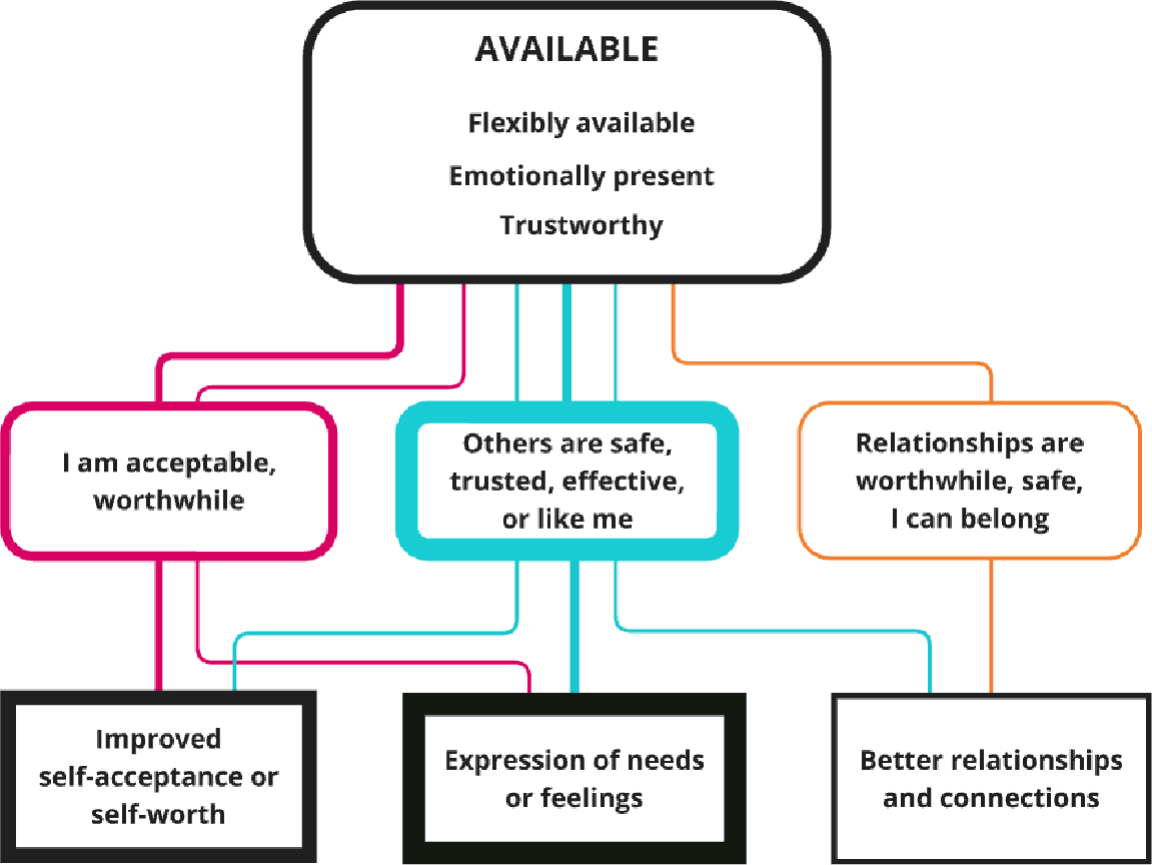
Nested CMOCs for the available interpersonal context. Represents the interpersonal factors included in the available context. The coloured soft rectangles represent the mechanisms, and hard rectangles are the outcomes. The thickness of borders and lines represents the number of times mechanisms and outcomes were coded or linked. CMOCs, context–mechanism–outcome configurations.

**Figure 6:**
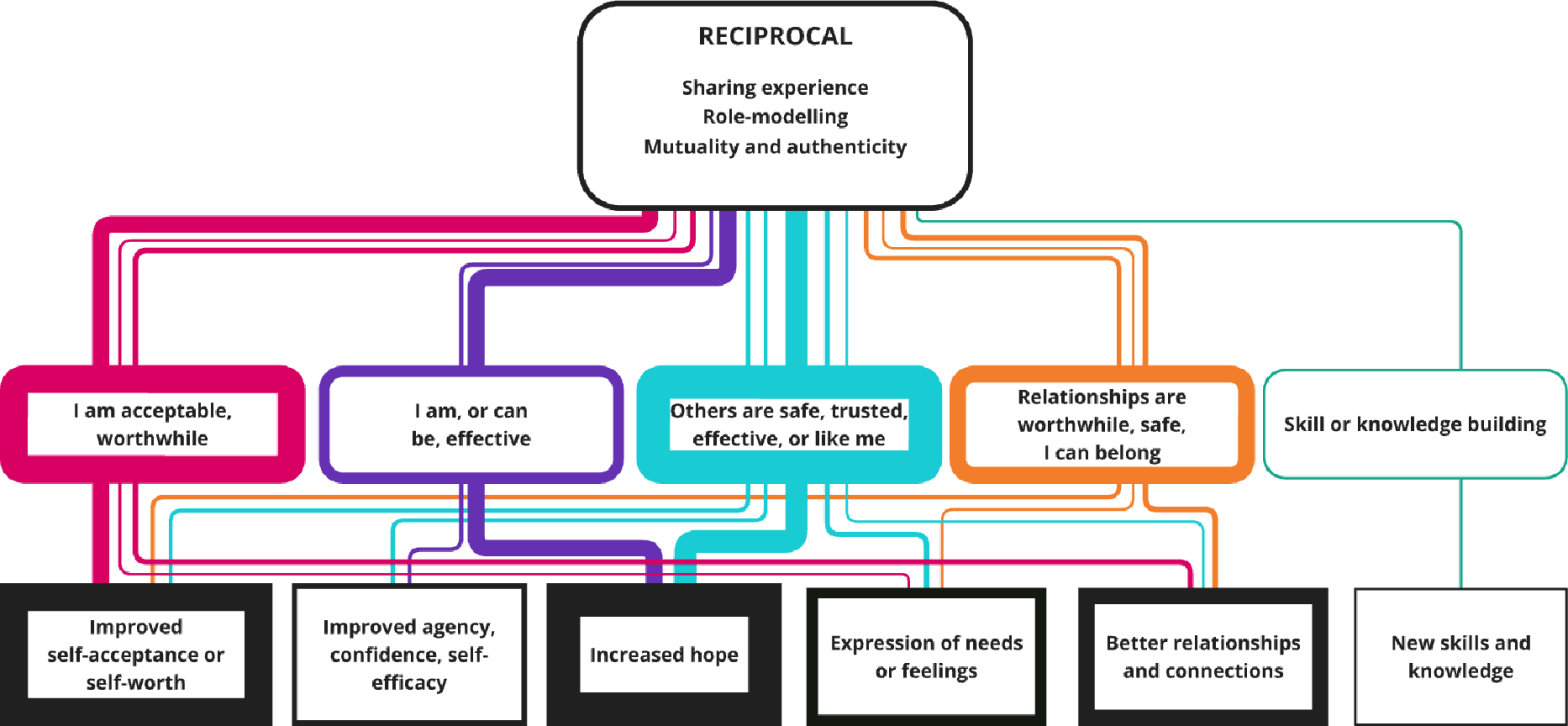
Nested CMOCs for the reciprocal interpersonal context. Represents the interpersonal factors included in the reciprocal context. The coloured soft rectangles represent the mechanisms, and hard rectangles are the outcomes. The thickness of borders and lines represents the number of times mechanisms and outcomes were coded or linked. CMOCs, context–mechanism–outcome configurations.

A stakeholder discussion was conducted with 15 peer support workers to consult on the resonance of initial programme theories (IPTs). This informed subsequent testing refinement and expansion components of the IPTs from published evidence. Context and outcome coding was largely inductive (directly extracted from the data). The coding of mechanisms was both inductive, deductive and retroductive.^17^ Where coding was deductive or retroductive, this was informed by substantive theory, discussions in the research team and with stakeholders.

### Substantive theory

Substantive theory is defined as theoretical approaches that are established in scientific literature. It is critical to understanding and contextualising mechanisms, which can involve deductive and retroductive reasoning.

The interpersonal contextual factors that were initially identified from the literature led to the identification of attachment as^18^,^20^ a key substantive theory. This iteratively informed the identification of reoccurring CMOCs in the data. Three attachment theory constructs were particularly influential: (1) secure base,^18^,^20^ (2) internal working models (IWMs)^17^,^19^ and (3) mentalisation.^21 22^ Constructs of personal recovery^23 24^ have been key to the development of mental health peer support roles and approaches^1 3^ and were key to coding, understanding and contextualising observed outcomes.

### Included studies

The final search strategy produced 13 220 results, following deduplication 9867 results remained. Data were extracted from 20 empirical articles. Of the studies included, 18 were qualitative and two were mixed methods (see figure 1).

### Patient and public involvement

This review was designed and delivered with people with lived experience of using mental health services and delivering peer support. This included the development of the review topic and questions, inclusion criteria, article selection, analysis process and development of outputs. This included a stakeholder group of 15 peer support workers (with lived experience of using services), who supported the refinement of the programme theory.

## Results

### Contextual factors

Five key contextual factors were identified for the peer relationship. These combine to form the APPEAR interpersonal framework: Accepting, Personalised Practice, Empowering, Available, Reciprocal. The infographics (figures 2–6) represent nested CMOCs identified in the papers.

### Accepting

The accepting interpersonal context (coded in 18 papers—see online supplemental table 4 S5 for the codes for each paper) describes a relationship defined by understanding and validation (coded in 16 papers), compassion and non-judgement (coded in 14 papers), feeling relaxed or comfortable (coded in 13 papers) and explicitly valuing the person and what they are expressing (coded in seven papers). Figure 2 shows the findings from nested CMOCs.

A non-judgemental and validating stance was linked to reduced social isolation, improved relationships and connections, and a better sense of belonging. This was most often via the causal mechanism of feelings of personal acceptability:^25^,^29^ ‘consumer peer workers can validate the unique experiences of BPD (borderline personality disorder) which are often not understood by others, helping consumers feel less judged and isolated. “They (consumers) don’t feel alone; they don’t feel weird or different. Last week*,* one of the girls said she doesn’t feel like she’s got two heads. She feels like she’s a normal kind of person and that she’s not being judged’.^25^^(p5)^ The stance and outcomes described above were also linked via the causal mechanisms of feelings that other people are safe, trusted or like me,^2529^,^31^ and that relationships are safe, worthwhile or that I can belong.^25 29 30^ Peer support stakeholders reflected that you can never truly know a person, but that curiosity is critical to attempting to understand people and their recovery needs.

An accepting relationship was also linked to better expression of needs and/or feelings, mainly via the contextual mechanism of feelings that other people are safe and can be trusted:^2728 31^,^33^ ‘I guess it’s that honesty and that trust, they do know what I’ve been through. They’re giving me that example and saying, I know where you’re coming from, that happened to me once in a shopping centre. I know straightaway they know, so I know I can open up more without being embarrassed, shot down, told no, it can’t be like that’.^32^^(p192)^ Feelings that I can be acceptable or even worthwhile were linked to the outcomes of expression of needs^27 31 32^ and improved self-worth.^4 30 34^

### Personalised practice

The interpersonal context of Personalised Practice (coded across 14 papers, see online supplemental table 4 S5) relates to the importance of a relationship that pivots on understanding the individual in their recovery (coded in eight papers) and supporting their individual needs within this (coded across 11 papers). Figure 3 the findings from nested CMOCs.

A relationship that pivots on understanding and supporting individual need was found to be linked to improved self-worth, most commonly via the causal mechanisms of feelings that I can be acceptable or worthwhile^31 32^ or that I can be effective in my recovery^26 31^ ‘… peer staff viewed their encouragement of participant interests as crucial to their role. In addition to reawakening hopes and providing experiences of pleasure, peer staff viewed these kinds of experiences as leading to the rediscovery (or in some cases, discovery) of dormant or latent abilities in the participants. Reclaiming or becoming aware of such abilities was viewed by the peers as one path to improving participants’ sense of self-esteem and self-worth’.^26^^(p312)^

The peer support worker stakeholder group identified that having individual needs recognised and met both creates feelings of personal acceptability and relational safety. This was reflected in the finding that a Personalised Practice relationship was also associated with the outcome domain of experiencing better relationships or connections. This was mainly via the causal mechanism of feelings that relationships are worthwhile, can be safe and/or a sense of belonging:^26 27^ ‘The majority of veteran participants valued how the PSW role connected them to the clinical and well-being support they needed, including the PSW drop-in service and social activities. Most participants (V, PSW & C) felt this helped to improve the veterans’ quality of life, and enhance their feelings of safety and social inclusion’.^27^^(p650)^

### Empowering

The interpersonal context of empowering (coded in 18 papers) was made up of the interpersonal factors of facilitating agency and control (coded in 15 papers), encouragement of recovery supporting activity (coded in seven papers), and the sharing of power or aiming for equality (coded in five papers). Figure 4 shows nested CMOCs.

The most common outcome linked to this relational context was improved agency, confidence and self-efficacy, via the causal mechanism of beliefs that I can be (or am) effective:^2628 31 35^,^39^ ‘Workers reported that they felt encouraging autonomy and person-centred alternatives to traditional care were beneficial for service-users as it encouraged greater levels of confidence amongst service-users in carrying out recovery-oriented tasks, and often resulted in better engagement with the service’^.31(p513)^ The peer support worker stakeholders questioned the framing of effectiveness, suggesting that this could instead be characterised as progressiveness, aligning with principles of change and growth in recovery.

This superordinate relational context was also linked to improved expression of need, but this was via a range of mechanisms including the belief that relationships are worthwhile.^26^ The ability to understand and label internal states,^36^ and the beliefs that others are effective,^32^ I can be effective,^36^ and I am acceptable or worthwhile.^32^ ^40^

### Available

The Available interpersonal context (coded in 13 papers) includes the themes of trustworthiness (coded in 11 papers), flexible availability (ie, contacts scheduled more flexibly to individual need) (coded in nine papers) and emotional presence (coded in seven papers). Figure 5 depicts the nested CMOCs.

The interpersonal context of Availability was most commonly associated with increased expression of needs and/or feelings, via the causal mechanism of beliefs that others are safe, effective or like me:^27 31 32^‘If you know they come from a similar place as yourself you’re more likely to open up a little bit more and relax a little bit more and trust a little bit more. I think you’ve got a little bit more trust in being a little bit more open to them … They’ve got a greater understanding of it, which is helpful knowing that someone’s not sitting there judging you or thinking I’m better than you or whatever’.^32^^.(p192)^

The available interpersonal context was linked to improved self-worth, mainly via the causal mechanism of feelings that I am acceptable or worthwhile.^4 27 34^ Finally, this superordinate interpersonal context was linked to improved relationships via the causal mechanism of belief that relationships can be safe and worthwhile:^30 31^*‘*… frequency of contact was critical in facilitating change. This factor was considered both important to relationship formation, but also in providing enough contact for participants to feel cared for during periods of crisis’.^31(p513)^ The peer support stakeholder group stressed the importance of boundaries and the maintenance of a professional relationship, stating that the key was balancing flexibility with boundaries, as too much availability was not practically possible and might undermine shared responsibility and empowerment.

### Reciprocal

The superordinate interpersonal context of reciprocity (coded in 19 papers) describes a relationship that values both peers and service users as contributors to the relationship. This was made up of the descriptive themes of the sharing of lived experience (coded in 18 papers), role-modelling of recovery (coded in 13 papers) and mutuality (defined as evidence of both parties benefitting from the relationship) (coded in 11 papers). Figure 6 depicts the nested CMOCs.

The peer support stakeholder group reflected the importance of reciprocity to enabling the expression of vulnerability. Sharing lived experience was identified as a key factor, and peer support stakeholders reflected that the way lived experience is shared is critical—emphasising that it should only be in response to the needs of the service user (rather than the needs of the peer support worker or in an attempt to establish credibility).

The outcome most commonly linked to a reciprocal relationship was hope, via the causal mechanism that others can be effective and are like me:^427 31 37 39 41^,^43^ ‘…mentees saw the peer worker relationship as different from ‘usual’ clinical interventions in terms of key items such as reciprocity and sharing. Strong themes emerged regarding the instillation of hope for recovery and a sense of agency; inspiration was gleaned from being able to access support from someone who had the lived experience of illness and of recovery’.^41^^(p7-8)^

Reciprocity was also linked to improved self-acceptance or self-worth, via the causal mechanisms of beliefs that I am acceptable or worthwhile^4 27 34 42^ and that relationships can be safe and/or worthwhile and/or that I can belong:^4 27^ ‘Many participants said it made them feel “normal”, that they belonged or were not alone, like Kate who said, “them just talking about their experiences was more of a help than I think a lot of … than they could imagine, ‘cause it made me realize there’s other people”’*.*^4^^(p.10)^

### Interactions and synergies between the superordinate themes

These interpersonal contexts or factors taken together form the Accepting, Personalised Practice, Empowering, Available and Reciprocal (APPEAR) framework. The framework includes five pivotal interpersonal contexts that interact to create the conditions for improved personal recovery outcomes in the domains of (1) self-acceptance, (2) confidence, (3) hopefulness, (4) self-expression, (5) relationships and (6) knowledge and skills. The findings map causal mechanisms underlying the observed context to outcome patterns. These outcomes are likely to be achieved as much via the interactions between contextual factors as the individual contexts themselves.

Additionally, there are likely perpetuating feedback loops between outcomes via the mechanisms in the framework. For example, confidence in self-management of recovery is likely to be consolidated by the accumulation of experiences of individual progress, growth and effectiveness. Equally, the belief that relationships can be safe and worthwhile is likely to result in a wider range of improved relationships, which in turn would consolidate the belief that relationships are safe and worthwhile. As such, personal recovery is a self-consolidating and self-perpetuating process. The APPEAR framework demonstrates how key interpersonal factors can create the initial conditions that support personal recovery, via a range of maintaining and perpetuating mechanisms.

## Discussion

Peer support is a well-recognised approach for supporting personal recovery and the nature of the relationship is understood to be a key component of this. What was not understood was how and why key relational contexts achieved these outcomes. Our synthesis identified pivotal relational factors, the outcomes these are linked to and the mechanisms of change underlying these configurations. The findings informed a framework to operationalise the delivery of interpersonal peer support approaches.

Attachment theory and models were identified as key substantive theory in initial and iterative CMOC constructions.^18^,^20^ Mental health difficulties have been empirically found to be linked to significant disruptions to early attachment relationships and the formation of a secure base.^44 45^ Positive attachment relationships in adulthood have been found to improve mental health,^46^ suggesting that the peer relationship may play a critical role in rescripting negative early attachment relationships. The APPEAR framework aligns with the attachment theory construct of a secure base. This refers to the concept that caregivers provide a consistent, safe and dependable foundation to support exploration, knowledge and skill development.^18^,^20^ The interpersonal factors of acceptance, person-centredness, empowerment, availability and reciprocity may create a safe base for exploration that is critical for personal recovery (eg, attending social groups and engaging in meaningful activity).

The peer worker relationship is adult-to-adult and time-limited. The emphasis in models of personal recovery on autonomy, agency and control is likely key to the transitional nature of peer support. This was emphasised by the peer worker stakeholders’ reflections on the importance of consistent boundaries, and prioritisation of independence and self-efficacy (although the term progressive was preferred). It is likely that interpersonal contexts such as acceptance, person-centredness and availability balanced with empowerment are key to the safe yet progressive nature of peer support interventions. Additionally, the sharing of lived experience and role-modelling of recovery facilitates reciprocity and mutuality, inspiring self-acceptance, hopefulness and a sense of belonging.

The secure base that peer support provides may offer a foundation for personal recovery, but (as with all attachment relationships) it is critical that the mechanisms of change are internalised to enable a process of personal recovery that is ultimately autonomous and independent from the relationship. Attachment theory suggests two key mechanisms for this: (1) the updating of IWMs and (2) mentalisation.

IWMs are mental representations of the self, others and relationships. They are cognitive frameworks comprising thoughts and feelings that guide expectations and interpretations.^18^,^20^ IWMs are initially formed in early caregiver relationships but can be developed and updated by significant attachment relationships throughout life^47^,^49^ including in therapy.^50 51^ In the APPEAR framework, thoughts about the self (eg, I am acceptable, worthwhile, progressive or effective in my recovery) others (eg, others are safe and can be effective in their recovery) and relationships (eg, that they can be safe, rewarding and worthwhile) could be conceptualised as IWMs that are updated by peer relationships to form new expectations for future relationships. In this way, the updated IWMs could support a longer term, self-perpetuating and process of recovery.

Mentalisation is the ability to understand internal mental states (such as thoughts and feelings) and interpret behaviour in terms of such underlying mental states.^21 22^ The development of mentalisation is thought to be a complex process influenced by early attachment relationships.^52 53^ Mentalisation is initially developed in early attachment relationships with caregivers. Key mechanisms are thought to include whether they are aware of and responsive to need, and the level of explicit reflection on their own and the child’s internal mental states.^52 53^ Early caregiver relationships are foundational, but the ability to mentalise both one’s own and other’s mental states has been found to be responsive to subsequent attachment relationships including therapy.^22 54^ In the APPEAR framework, understanding and labelling feelings and needs (both one’s own and others’) was an important mechanism of change for expression of needs, improved confidence and self-worth, and improved relationships. The ability to mentalise, alongside feelings and expectations that one is worthwhile and effective, and that relationships can be safe and worthwhile, could contribute to better self-regulation of emotions and improved relationships.

### Strengths and limitations

This synthesis integrated service user and staff perspectives to develop an evidence-based framework for understanding the mechanisms of change associated with the key relational components of peer support. This should support training, supervision and delivery of peer support services. This was developed from the synthesis of the current evidence base. The resulting framework would benefit from direct empirical testing and refinement.

The final search for this study was conducted in May 2024 and does not include articles published after this. Qualitative meta-synthesis prioritises conceptual and theoretical insight over exhaustive inclusion of the evidence,^55^ but the lack of the most recent evidence is a limitation of the current review. In accordance with realist review methodology, inclusion of articles was based on realist assessments of relevance and rigour to theory building and refinement,^13^ rather than a formal appraisal of quality. Formal assessments of rigour using rating scales, as commonly practised in systematic reviews, are not suitable for theory-driven reviews. In realist analysis, which is iterative and incorporates the perspectives of people with content, lived and professional expertise, evaluations of the trustworthiness and credibility of resulting programme theories are embedded throughout the process.^13^ Although ratings scales might have classified the quality of some data as ‘low’, the collective insights from the included studies contributed to a conceptually robust programme theory ready for further testing.

This synthesis did not actively incorporate findings or develop theory regarding unintended or potential negative outcomes such as those identified by a systematic review and meta-analysis of group peer support interventions.^56^ This represents a limitation, as such insights could have provided a more comprehensive understanding of when and why peer support interventions may fail to achieve their intended effects or result in adverse outcomes. We acknowledge this gap and recommend future research to explicitly examine potential unintended consequences or harms to inform the development of more nuanced programme theory. This synthesis also did not include the specific types of activities that are undertaken to achieve personal recovery. Additionally, despite the endorsement of peer support by the WHO, the studies included in the current review were mainly conducted in high-income countries including Europe, USA, Canada and Australia, with one study conducted in China^29^ and one in Singapore.^37^ This limits transferability of the resulting framework to contexts where there may be important cultural differences in key interpersonal mechanisms and where there are differences in mental healthcare delivery. Future research could expand the transferability of the findings from the current synthesis.

## Conclusion

The APPEAR framework offers a useful reflective tool for the training and delivery of mental health peer support. The contextual factors, causal mechanisms and outcomes are presented in the framework as distinct factors. The reality is, of course, more nuanced, interactive and complex. The APPEAR framework was developed from a literature synthesis with stakeholder input and would benefit from empirical testing and refinement to better understand the complexity within the peer relationship.

The APPEAR framework can help support workers, service users and the mental health system more broadly to understand how to create the interpersonal conditions to support personal recovery. This is best understood within an attachment model, whereby the peer-to-peer relationship creates the initial conditions for the development or consolidation of psychological mechanisms that support a self-perpetuating process of recovery that is ultimately independent from the relationship. The aim of the APPEAR framework is to provide a foundation for understanding and developing peer support interventions to improve mental health and personal recovery outcomes.

## Supplementary material

10.1136/bmjopen-2025-105211 online supplemental file 1

10.1136/bmjopen-2025-105211 online supplemental table 1

10.1136/bmjopen-2025-105211 online supplemental table 2

10.1136/bmjopen-2025-105211 online supplemental table 3

10.1136/bmjopen-2025-105211 online supplemental table 4

10.1136/bmjopen-2025-105211 online supplemental table 5

## Data Availability

All data relevant to the study are included in the article or uploaded as supplementary information.
