# Supplementary material for: Mental health peer support relationship: a realist informed qualitative meta synthesis
Source: BMJ Open. 2025 Dec 30;15(12):e105211. doi: 10.1136/bmjopen-2025-105211 (PMC12766804; doi:10.1136/bmjopen-2025-105211)
Supplement: online supplemental file 1 [file bmjopen-15-12-s001.docx]

**Appendix 1 – full search strategy**

The search strategy was as follows (adjusted appropriately for individual databases):

1. Title and abstract (TIAB) "peer work" OR "peer specialist" OR "peer navigator*" OR "peer exper*" OR "peer provi*" OR "lived experience" OR "lived expertise*" OR "expert by experience" OR "mutual support" OR "consumer provi*" OR "service user work*" OR "expert peer*" OR "peer support*"
2. TIAB ("consumer participation*" OR "peer group") OR mainsubject.exact ("Peer Counselling")
3. Combine 1 & 2 with OR
4. TIAB mental* OR schiz* OR depress* OR "obsessive compulsive disorder*" OR OCD OR "eating disorder*" OR anorexi* OR bulimi* OR bipolar OR psych* OR PTSD OR BPD OR anxi* OR "personality disorder*" OR identity disorder* OR "mood disorder*" OR "panic disorder*" OR phobi* OR "affective disorder*"
5. mainsubject.exact ("mental health services") OR TIAB ("psychiatric care")
6. Combine 4 & 5 with OR
7. Combine all together with AND
