# Supplementary material for: Mental health peer support relationship: a realist informed qualitative meta synthesis
Source: BMJ Open. 2025 Dec 30;15(12):e105211. doi: 10.1136/bmjopen-2025-105211 (PMC12766804; doi:10.1136/bmjopen-2025-105211)
Supplement: online supplemental table 1 [file bmjopen-15-12-s002.docx]

**Table 1. Study and sample demographics.**

| Paper | Title | Country | Sample Size | Sample | Mental Health Condition | Service |
| --- | --- | --- | --- | --- | --- | --- |
| Barr et al (2020)^25^ | Using peer workers with lived experience  to support the treatment of borderline  personality disorder: a qualitative study of  consumer, carer and clinician perspectives | Australia | 36 | 5 consumers, 7 consumer peer workers, 6 carers, 6 carer peer workers and 12 mental health professionals | Borderline personality disorder (BPD) | Individuals from services and locations which provide peer support to consumers with BPD or their carers were invited to participate, and participants were asked to identify other potential participants |
| Beveridge et al (2019)^41^ | Peer mentoring for eating disorders: results from the evaluation of a pilot program | Australia | 30 | Mentees. | Eating disorders | 12 participants were recruited from the Inpatient Service at the Austin Hospital, 14 from the Day Patient Program at St Vincent’s Hospital (the Body Image & Eating Disorders Treatment & Recovery Service; BETRS) and 4 from The Melbourne Clinic Inpatient Service. |
| Bradstreet (2010)^39^ | Developing peer support  worker roles: reflecting  on experiences in Scotland | Scotland | Not Reported | Not Reported | Not reported | Not reported |
| Gidugu et al (2020)^4^ | Individual Peer Support: A Qualitative Study of Mechanisms of its Effectiveness | United States | 26 | Adults with a psychiatric condition who have had 10 sessions of individual peer support | Individuals with psychiatric conditions | A local behavioural healthcare organisation. |
| Gillard et al (2015)^42^ | Developing a change model for peer worker interventions in mental health services: a qualitative research study | United Kingdom | 71 | 3 aged 18-25 10 aged 26-35 26 aged 36-45 14 aged 46-55 10 aged 56-65 2 over the age of 65 | General adult mental health and adult personality disorders | National Health Service mental health trusts, peer-led organisations and voluntary sector organisations |
| Gray et al. (2017)^33^ | Finding the right connections: Peer support within a community based mental health service | Australia | 19 | Peer and non-peer staff | Not reported | Community-based mental health services. |
| Gruhl, LaCatre & Calixte (2016)^43^ | Authentic peer support work: challenges and opportunities for an evolving occupation | Canada | 51 participants in the survey,  33 participants in the focus group,  1 in the interview | Peer support workforce | Not reported | Mainstream mental health services. |
| Hurley et al (2018)^32^ | Qualitative study of peer workers within the ‘Partners in Recovery’ programme in regional Australia | Australia | 22 | 4 Peer workers 4 managers 4 managers 10 support facilitators | Severe and persistent mental illness and complex needs | A Partners in Recovery programme |
| Mourra et al. (2014)^26^ | Pushing, Patience, and Persistence: Peer Providers' Perspectives on Supportive Relationships | United States | 7 | Peer Staff | Serious mental illness | Psychiatric admission to an inpatient setting within the previous 18 months |
| Ng and Barlas (2023)^37^ | A bridge to recovery: an interpretative phenomenological analysis with peer  support specialists in Singapore | Singapore | 6 | Mental health peer support specialists | Psychiatric conditions | A mental healthcare organisation in Singapore |
| Nossek et al (2021)^35^ | Evolvement of Peer Support Workers’ Roles in Psychiatric Hospitals:  A Longitudinal Qualitative Observation Study | Germany | 30 observations | Open nonparticipant observations of 25 multiprofessional team meetings and 5 transregional peer support worker meetings | Psychiatric conditions | Three adult psychiatric hospitals in North Rhine-Westphalia, Germany |
| Otte et al. (2020)^40^ | Beneficial effects of peer support in psychiatric hospitals. A critical reflection on the results of a qualitative interview and focus group study | Germany | Total number of participants unspecified | Eight peer support workers were interviewed. Five focus groups with mental health professionals (total n unspecified) and 1 focus group with 5 PSWs. | Psychiatric conditions | Five adult psychiatric hospitals, which belong to the Psychiatry Network of the Regional Association of Westphalia-Lippe in Germany |
| Scanlan, Hancock & Honey (2017)^30^ | Evaluation of a peer-delivered, transitional and post-discharge support program following psychiatric hospitalisation | Australia | 17 | Consumers of a peer support trial | Severe mental illness | Post discharge (from inpatient psychiatric admission) support programme. |
| Storm (2020)^36^ | Peer Support in Coordination of Physical Health and Mental Health Services for People With Lived Experience of a Serious Mental Illness | United States | 28 (23 mental health professionals and 5 peer support specialists) | Peer support workers and mental health professionals | Not reported | Community mental health centres |
| Tse et al. (2017)^29^ | A one-year longitudinal qualitative study of peer support services in a non-Western context: The perspectives of peer support workers, service users, and co-workers | China | 34 | 5 Peer support worker trainees, 14 service users and 14 co-workers | Schizophrenia or depression | Four partnered non-governmental organizations offering peer services. |
| Van Zanden & Bliokas (2022)^31^ | Taking the Next Step: A Qualitative Study Examining Processes of Change in a Suicide Prevention Program Incorporating Peer-Workers | Australia | 11 | Nearly half (4) of the participants were under the age of 35. Peer workers were more likely to be older in age and male (67% male) while clinicians were more likely to be younger and female (80% female) | Individuals over 16 who have presented to local Emergency Departments following a suicide attempt or because of high risk for suicide | Recruited from a suicide prevention program locally known as "*Next Steps*" (follow up aftercare service for individuals over 16 who have presented to local Emergency Departments following a suicide attempt or because of high risk for suicide. This is a non-government organization. |
| Wall et al (2022)^34^ | Experiences and Challenges in the Role as Peer Support Workers in a Swedish Mental Health Context - An Interview Study | Sweden | 10 | Not Reported | Different experiences of severe mental illness such as bipolarity, psychosis, emotional instability, anxiety and depression | Currently or previously employed in outpatient or inpatient settings within general adult psychiatric or psychosis care. |
| Walker et al (2024)^28^ | A theory of change for one-on-one peer support for older adolescents and  young adults | United states | 52 | 35 peer support specialists (PSS) and 17 peer support users (PSU) | Services for first episode psychoses  and young people with serious mental health conditions. | Young adults who had provided (PSS) and/or participated (PSU) in peer support as a part of formal services provided in community-based outpatient programs focused on serving young people diagnosed with serious mental health conditions in the United States. |
| Walsh et al (2018)^38^ | Understanding paid peer support in mental health | Australia | 32 | 21 peers participated in focus groups, 4 managers/ coordinators. 3 paid peer support workers and 4 other stakeholders | Not reported | Two Queensland-based, consumer-operated organizations offering paid peer support programmes. Organisation A was a regional centre that included a peer support telephone line and was part of a larger organisation whereas the second was an urban centre that provided residential and community-based support and reported directly to a board |
| Weir et al (2019)^27^ | Military veteran engagement with mental health and well-being services: a qualitative study of the role of the peer support worker | Scotland | 18 | Consumers/vets: 3 aged 25 - 34 7 aged 25 - 54 PSWs: Aged between 35 and 54 Clinicans: Not Reported | Not reported | NHS veteran mental health and wellbeing clinic |
