# Supplementary material for: Mental health peer support relationship: a realist informed qualitative meta synthesis
Source: BMJ Open. 2025 Dec 30;15(12):e105211. doi: 10.1136/bmjopen-2025-105211 (PMC12766804; doi:10.1136/bmjopen-2025-105211)
Supplement: online supplemental table 3 [file bmjopen-15-12-s004.docx]

**Table 3, RAMESES publication standards checklist**

| **Item no** | **Standard** | **Reported on page #** |
| --- | --- | --- |
| 1 | Title identified as realist review | P.1 Title ‘realist informed’ |
| 2 | Abstracts contains brief details of the study’s background, review question or objectives; search strategy; methods of selection, appraisal, analysis and synthesis of sources; main results; and implications for practice. | P. 2-3 Abstract |
| 3 | Explain why the review is needed and what it is likely to contribute to existing understanding of the topic area. | P. 2-3 Introduction |
| 4 | State the objective(s) of the review and/or the review question(s). Define and provide a rationale for the focus of the review | P.3 Objectives |
| 5 | Any changes made to the review that was initially planned should be briefly described and justified. | n/a |
| 6 | Explain why realist synthesis was considered the most appropriate method to use. | P.2-3 Introduction and P.3 Method |
| 7 | Describe and justify the initial process of exploratory scoping of the literature. | No |
| 8 | State and provide a rationale for how the iterative searching was done. Provide details on all the sources accessed for information in the synthesis. For example, where electronic databases have been searched, details should include, for example, the name of the database, search terms, dates of coverage and date last searched. If individuals familiar with the relevant literature and/or topic area were contacted, indicate how they were identified and selected. | P.3-4 Search strategy and selection criteria |
| 9 | Explain how judgements were made about including and excluding data from documents and justify these. | P. 3-4 Search strategy, selection criteria and P. 4-5 Data extraction and Coding and synthesis |
| 10 | Describe and explain which data or information were extracted from the included documents and justify this selection. | P.3-4 Search strategy and selection criteria |
| 11 | Describe the analysis and synthesis processes in detail. This section should include information on the constructs analysed and describe the analytic process. | P.4-5 Coding and synthesis and P.5 Substantive theory |
| 12 | Provide details on the number of documents assessed for eligibility and included in the review with reasons for exclusion at each stage, as well as an indication of their source of origin (for example, from searching databases,reference lists and so on). | P.4 Figure 1 PRISMA flow diagram |
| 13 | Provide information on the characteristics of the documents included in the synthesis. | S.2 Table 1 – demographics of included studies |
| 14 | Present the key findings with a specific focus on theory building and testing. | P.6-11 Results |
| 15 | Summarise the main findings, taking into account the synthesis’ objective(s), research question(s), focus and intended audience(s). | P.10 Interactions and synergies between the superordinate themes and P.10-12 Discussion |
| 16 | Discuss both the strengths of the review and its limitations. These should include (but need not be restricted to) (a) consideration of all the steps in the synthesis process and (b) comment on the overall strength of evidence supporting the explanatory insights that emerged. The limitations identified may point to areas where further work is needed. | P.11-12 Strengths and limitations |
| 17 | Where applicable, compare and contrast the synthesis’ findings with the existing literature (for example, other reviews) on the same topic. | n/a |
| 18 | List the main implications of the  findings and place these in the context  of other relevant literature. If  appropriate, offer recommendations for  policy and practice. | P.12-13 Conclusion |
| 19 | Provide details of funding source (if any) for the synthesis, the role played by the funder (if any) and any conflicts of interests of the reviewers. | P.13 Funding |
