# Supplementary material for: Mental health peer support relationship: a realist informed qualitative meta synthesis
Source: BMJ Open. 2025 Dec 30;15(12):e105211. doi: 10.1136/bmjopen-2025-105211 (PMC12766804; doi:10.1136/bmjopen-2025-105211)
Supplement: online supplemental table 4 [file bmjopen-15-12-s005.docx]

**Table 4. Codes and themes for each article**

| Paper | **Interpersonal context (number of codes)** | | | | | **Mechanisms** | | | | | | **Outcomes** | | | | | |
| --- | --- | --- | --- | --- | --- | --- | --- | --- | --- | --- | --- | --- | --- | --- | --- | --- | --- |
|  | Accept | Person-pivoting | Empower | Available | Reciprocal | I am Acceptable or worthwhile | I am/could be Effective | Others safe, trusted, effective, like me | Connection/ sense of belonging | Understand and label mental states | Knowledge and skill development | Self-worth | Agency, confidence, self-efficacy | Hope | Expression of feelings and/or needs | Better relationships and connections | New skills/knowledge |
| Barr et al, (2020)^25^  5 CMOCs | VD (5), NJ (1), CM (3) | SU (1),  IU (4) | AG (1) | TW (1), EA (2) | SH (2), RM (2), MU (1) | XX | X | X | X |  | XX |  | X | X | X | XX | XX |
| Beveridge (2019)^41^  1 CMOC | NJ (2), CM (2), VU (1) |  | EC (1), EQ (1) |  | SH (3), MU (1) | X |  | X |  |  |  |  | XX | XXX |  | XX |  |
| Bradstreet (2010)^39^  0 CMOCs |  |  | AG (1), EC (1) |  | SH (1), RM (2), MU (1) |  |  |  |  |  |  |  | X | X |  |  | X |
| Gidugu (2020)^4^  4 CMOCs | VD (1), CM (2), VU (1) | IU (2) | AG (1), EC (3) | EA (7) | SH (6), RM (2), MU (1) | XXX | XX | XXXX | XX |  |  | XXX | XXX | XXX | X | XXX |  |
| Gillard (2015)^42^  6 CMOCs | VD (4), CM (1) | SU (1) | EC (1) | TW (1) | SH (3), RM (4) | XX | X | XXXX |  |  |  | XX | XX | XX | XXXX | XX |  |
| Gray (2017)^33^  0 CMOCs | VD (1),  NJ (3), CM (1), |  | AG (1), EQ (1) | TW (2), PA (2) | SH (1), RM (1), MU (1) |  |  | XX |  |  |  |  |  | X | XX |  |  |
| Gruhl (2016)^43^  1 CMOC | VD (2), NJ (1), | SU (2),  IU (1) |  |  | SH (1), RM (3), MU (2) |  |  | XX |  |  |  |  |  | X |  |  |  |
| Hurley (2018)^32^  3 CMOCs | VD (1), NJ (2), CM (2) |  | AG (1), EQ (1) | TW (1) | SH (1), RM (1) | X |  | XX |  |  |  |  |  |  | XX |  |  |
| Mourra (2014)^26^  7 CMOCs | VD (2), NJ (2), VU (1), | SU (3),  IU (4) | AG (3), EC (5) |  | SH (1) | XX | XXXXX | X | XX | X | X | XXX | XXXXX |  | X | XXX |  |
| Ng  (2023)^37^  2 CMOCs | VD (1)  NJ (1) | SU (1) | EC (2) |  | SH (1) |  | X | X |  |  |  |  | X | X |  |  |  |
| Nossek (2021) ^35^  0 CMOCs | VD (1), CM (1), VU (1) |  | AG (1) | PA (2) |  |  |  |  |  |  |  |  |  | X |  |  |  |
| Otte (2019)^40^  3 CMOCs | VD (4), NJ (1), CM (1) | IU (2) | AG (1) | TW (3), EA (1), PA (4), VD (1) | SH (1), RM (1) | XXX | XX | X | X |  |  |  | X | X | X |  |  |
| Scanlan (2017)^30^  4 CMOCs | VD, (3), NJ (2), CM (2), VU (3) | SU (3) | AG (2) | TW (1), EA (1), PA (1) | RM (2) | X | X | XXX | XX |  |  | X | X | XXX | X | XXX | X |
| Storm (2020)^36^  1 CMOC |  | SU (5) | AG (4) |  | SH (2), MU (1) |  | X |  |  | X | X |  | X |  | X |  |  |
| Tse (2017)^29^    2 CMOCs | VD (2), CM (1), |  |  | TW (1), PA (1) | SH (1) | X |  | X | X |  |  |  |  |  |  | XX |  |
| Van Zanden (2022)^31^  7 CMOCs | VD (2), NJ (1), CM (2), VU (2) | SU (4)  IU (1) | AG (3), EC (3) | TW (3), EA (2), PA (2) | SH (2), RM (4) | XXXXX | XXXXX | X | X | X | X | X | XXXXX | XXXXXX | XX | X | X |
| Walker (2024)^28^  4 CMOCs | VD (4)  NJ (3) | SU (2) | AG (5) |  | SH (6)  RM (1)  MU (2) | XXX | XXX | XXX | XX |  |  |  | XX | XX | XXX | XX |  |
| Wall (2022)^34^  3 CMOCs | VD (1), NJ (3), CM (2), VU (2) | SU (2) | AG (2), EQ (1) | TW (2), EA (1), PA (1) | SH (3), RM (2), MU (3) | XXX |  | XXXX | XX |  |  | XX |  | XXX |  |  |  |
| Walsh (2018)^38^  1 CMOC | NJ (4), CM (2) | IU (2) | AG (3), EQ (1) | TW (1), EA (2), PA (2) | SH (6), MU (1) |  | X |  |  |  |  |  | X |  |  | X |  |
| Weir (2019)^27^  6 CMOCs | VD (4),  NJ (8), CM (3), | SU (3),  IU (6) | AG (1) | TW (3),  PA (2) | SH (4), RM (2), MU (1) | XXXXX | X | XX | XXX | X |  | XXX |  | X | XX | XX |  |

**Codes for interpersonal context**

Accepting: Understanding and validating (VD), Compassionate and non-judgemental (NJ), Comfortable/relaxed/ informal (CM), Valuing (VU).

Person-pivoting: Supportive of individual need (SU), Individualised understanding (IU).

Empowering: Facilitating agency and control (AG), Encouraging (EC), Equality (EQ).

Available: Trustworthy (TW), Emotionally available, present (EA), Physically available (PA).

Reciprocal: Sharing experience (SH), Role modelling (RM), Mutuality and authenticity (MU).
