# Supplementary material for: Mental health peer support relationship: a realist informed qualitative meta synthesis
Source: BMJ Open. 2025 Dec 30;15(12):e105211. doi: 10.1136/bmjopen-2025-105211 (PMC12766804; doi:10.1136/bmjopen-2025-105211)
Supplement: online supplemental table 5 [file bmjopen-15-12-s006.docx]

**Table 5, Data examples from included articles with CMOC coding.**

| Paper, page number, type of data | Quote | CMOC |
| --- | --- | --- |
| Hurley et al, (2018)^32^ Page 192. First order data | I guess it’s that honesty and that trust, they do know what I’ve been through. They’re giving me that example and saying, I know where you’re coming from, that happened to me once in a shopping centre. I know straightaway they know, so I know I can open up more without being embarrassed, shot down, told no, it can’t be like that. That’s how a professional can be because they can say no, that doesn’t happen because it’s not there in the book. | Context: **Accepting** (understanding and validating)  Mechanism: Others are safe  Outcome: Expression of feelings and needs |
| Barr et al, (2020)^25^ Page 5. First and second order data | … consumer peer workers can validate the unique experiences of BPD [borderline personality disorder] which are often not understood by others, helping consumers feel less judged and isolated. “They (consumers) don’t feel alone., they don’t feel weird or different Last week one of the girls said she doesn’t feel like she’s got two heads. She feels like she’s a normal kind of person and that she’s not being judged”. | Context: **Accepting** (understanding and validating)  Mechanism: I am acceptable  Outcome: Better relationships and connections |
| Van Zanden et al, (2022)^31^ Page 513. Second order data | Peer-workers and clinicians appeared to regard highlighting strengths and clarifying values and goals as a critical factor in reducing feelings of hopelessness. Discussing strengths was regarded as a powerful tool in restoring feelings of self-worth and hope for the future. | Context: **Person-pivoting** (understanding the individual)  Mechanism: I am/can be effective  Outcomes: improved self-worth and hope. |
| Weir et al, (2019)^27^ Page 5. Second order data | The majority of veteran participants valued how the PSW role connected them to the clinical and well-being support they needed, including the PSW drop-in service and social activities. Most participants (V, PSW & C) felt this helped to improve the veterans’ quality of life, and enhance their feelings of safety and social inclusion. | Context: **Person-pivoting** (supporting individual need)  Mechanism: Relationships are worthwhile  Outcome: Better relationships, belonging, connection |
| Weir et al, (2019)^27^ Page 6. Second order data | the PSW role helped to provide veteran patients with a more veteran centred clinical process and treatment experience, and create an environment of belonging, shared identity and understanding | Context: **Person-pivoting** (supporting individual need)  Mechanism: Relationships are worthwhile  Outcome: Better relationships, belonging, connection |
| Mourra et al, (2014)^26^ Page 312.  Second order data. | peer staff viewed their encouragement of participant interests as crucial to their role. In addition to reawakening hopes and providing experiences of  pleasure, peer staff viewed these kinds of experiences as leading to the rediscovery (or in some cases, discovery) of dormant or latent abilities in the participants. Reclaiming or becoming aware of such abilities was viewed by the peers as one path to improving participants’ sense of self-esteem and self-worth | Context: **Person-pivoting** (supporting individual need)  Mechanism: I am effective  Outcome: Better self-worth |
| Mourra et al, (2014)^26^ Page 313.  Second order data. | Peer staff believed that, by recognizing participants’ abilities and talents, they could help improve the sense of self-esteem and self-confidence that participants would need in order to take next steps or try new activities in their pursuit of recovery. | Context: **Empowering** (encouraging)  Mechanism: I am effective  Outcome: improved self-worth |
| Gidugu et al, (2020)^4^ Page 9. Second order data. | Nearly all of the participants described receiving emotional support including helping them stay motivated and hopeful when they felt like giving up, saying things that built their self-esteem, conveying respect (M) and providing encouragement to work through their challenges. | Context: **Empowering** (encouraging)  Mechanism: I am acceptable  Outcome: improved self-worth. |
| Van Zanden et al, (2022)^31^ Page 153. Second order data. | Workers reported that they felt encouraging autonomy and person-centered alternatives to traditional care were beneficial for service-users as it encouraged greater levels of confidence amongst service-users in carrying out recovery-oriented tasks, and often resulted in better engagement with the service. | Context: **Empowering** (facilitating agency)  Mechanism: I am effective  Outcome: improved agency |
| Hurley et al, (2018)^32^ Page 192. First order data. | If you know they come from a similar place as  Yourself you’re more likely to open up a little bit more and relax a little bit more and trust a little bit more. I think you’ve got a little bit more trust in being a little bit more open to them… They’ve got a greater understanding of it, which is helpful knowing that someone’s not sitting there judging you or thinking I’m better than you or whatever. | Context: **Available** (trustworthy).  Mechanism: others are safe.  Outcome: Expression of needs or feelings. |
| Van Zanden et al, (2022)^31^ Page 153. Second order data. | …frequency of contact was critical in facilitating change. This factor was considered both important to relationship formation, but also in providing enough contact for participants to feel cared for during periods of crisis. | Context: **Available** (flexible availability).  Mechanism: I am acceptable.  Outcome: Better relationships. |
| Beveridge et al, (2019)^41^ Pages 7 & 8. Second order data. | …mentees saw the peer worker relationship as different from ‘usual’ clinical interventions in terms of key items such as reciprocity and sharing. Strong themes emerged regarding the instillation of hope for recovery and a sense of agency; inspiration was gleaned from being able to access support from someone who had the lived experience of illness and of recovery. | Context: **Reciprocal** (sharing experience)  Mechanism: Others are safe/like me  Outcomes: Hope and agency. |
| Gillard et al, (2015)^42^ Page 440. First order. | …so breaking down the stigma, it’s a slow and  cautious process…but by being open and when people see that when you’re well you just act normally . . it’s only when they’re unwell that their behaviour might seem strange but the rest of the time they’re just normal people… | Context: **Reciprocal** (role modelling).  Mechanism: others are effective, safe, like me.  Outcome: improved self-worth |
| Gidugu (2020)^4^  P10. First and second order | Many participants said it made them feel “normal,” that they belonged or were not alone, like Kate who said, “them just talking about their experiences was more of a help than I think a lot of . . . than they could imagine, ‘cause it made me realize there’s other people.” | Context: **Reciprocal** (sharing).  Mechanism: others are effective, safe, like me.  Outcome: improved self-worth |

First order data: direct participant quotes. Second order data: authors interpretations.
