# Supplementary material for: Mental health peer support relationship: a realist informed qualitative meta synthesis
Source: BMJ Open. 2025 Dec 30;15(12):e105211. doi: 10.1136/bmjopen-2025-105211 (PMC12766804; doi:10.1136/bmjopen-2025-105211)
Supplement: Supplementary file 7 [file bmjopen-2025-105211.reviewer_comments.pdf]

PEER REVIEW HISTORY

BMJ Open publishes all reviews undertaken for accepted manuscripts. Reviewers are asked to complete a checklist review form and are provided with free text boxes to elaborate on their assessment. These free text comments are reproduced below.

ARTICLE DETAILS

Title (Provisional)

The mental health peer support relationship: a realist informed qualitative meta synthesis.

Authors

Hackmann, Corinna; Green, Amanda; Pease, Ruby; Clegg, Izobel; Handley, Melanie

---

VERSION 1 - REVIEW

**Reviewer**	**1**
**Name**	**Thomacos, Nikos**
**Affiliation**	**RMIT University, Psychology Discipline**
**Date**	**10-Sep-2025**
**COI**	**None**

It was a pleasure to read this review, as it represents a methodologically rigorous, well-articulated, theory-driven synthesis of mental health peer support using a realist-informed qualitative methodology. The synthesis is robust, with clear articulation of how qualitative evidence was translated into themes and analytical constructs, culminating in the development of the APPEAR framework. A realist-informed approach is an appropriate approach for such a review, and the inclusion of stakeholder input enhances credibility, validity, and practical relevance of review.

Several areas for improvement should be considered to enhance the paper:

- The rigour assessment for the included studies was not detailed; inclusion was based on relevance rather than formal appraisal of methodological quality. This may result in studies of varying rigour being included, thus potentially affecting confidence in the findings. Suggest that this be considered and noted as a limitation.
- Use of CMOC (Context-Mechanism-Outcome Configurations) and retroductive theorising is appropriate, but it would be good to have some information about how raw data were modelled into these theoretical configurations, and how competing explanations were considered or ruled out.
- While the included papers may not have included anything regarding the unintended or negative outcomes of peer support, this is an important issue when considering the effects and impact of support groups. Moreover, realist syntheses can be more complete and thus impactful when they address challenges or situations where interventions may not work as intended and/or where negative outcomes are noted and acknowledged. It would therefore be good to have this issue identified in the discussion, at least.

Example papers about this issue:

Lyons, N., Cooper, C. & Lloyd-Evans, B. (2021). A systematic review and meta-analysis of group peer support interventions for people experiencing mental health conditions. *BMC Psychiatry*, *21*, 315. https://doi.org/10.1186/s12888-021-03321-z

Galinsky, M. J., & Schopler, J. H. (1994). Negative experiences in support groups. *Social work in health care*, *20*(1), 77–95. doi.org/10.1300/J010v20n01_09

Palant, A., & Himmel, W. (2019). Are there also negative effects of social support? A qualitative study of patients with inflammatory bowel disease. *BMJ Open*; *9*: e022642. doi: 10.1136/bmjopen-2018-022642

Reviewer **2**

Name **Chidarikire, Sherphard**

Affiliation **The University of Newcastle Australia, School of Nursing and Midwifery**

Date **15-Oct-2025**

COI **None**

Thank you for the opportunity to review this important study examining how key relational factors contribute to observed outcomes in mental health peer support. This is one of the best and most well-written papers I have reviewed. The authors are to be commended for their thoroughness and attention to detail, particularly in the methods section, which is explained clearly and comprehensively.

See feedback for the different sections of the manuscript below:

Title
No issues, title is good

Abstract
No issues

Introduction
The introduction is well written. No further comments. Well done.

Methods
A very comprehensive methods section including participants and procedures. Well done.

Results
Well-presented and very detailed, well done.

Discussion
The discussion is well presented with limitations of the study clearly highlighted.

Conclusion
No issues, well done

VERSION 1 - AUTHOR RESPONSE

Responses to reviewer's comments

Thank you for reviewing this manuscript and for the helpful feedback and comments. We have outlined how these were addressed (where this was required) below.

Reviewer 2

- Comment: The rigour assessment for the included studies was not detailed; inclusion was based on relevance rather than formal appraisal of methodological quality. This may result in studies of varying rigour being included, thus potentially affecting confidence in the findings. Suggest that this be considered and noted as a limitation. Response: this has been considered and noted as a limitation in the 'limitations' section of the manuscript (p13).

- Comment: Use of CMOC (Context-Mechanism-Outcome Configurations) and retroductive theorising is appropriate, but it would be good to have some information about how raw data were modelled into these theoretical configurations, and how competing explanations were considered or ruled out. Response: More information has been added to the 'coding and analysis' section on how theoretical configuration were achieve – including how competing explanations were ruled out (p6).

Comment: While the included papers may not have included anything regarding the unintended or negative outcomes of peer support, this is an important issue when considering the effects and impact of support groups. Moreover, realist syntheses can be more complete and thus impactful when they address challenges or situations where interventions may not work as intended and/or where negative outcomes are noted and acknowledged. It would therefore be good to have this issue identified in the discussion, at least. Response: Thank you for this. A discussion of this has been added to the 'limitations' section where this has been noted as a limitation that warrants further research (p13).

VERSION 2 - REVIEW

**Reviewer**	**1**
**Name**	**Thomacos, Nikos**
**Affiliation**	**RMIT University, Psychology Discipline**

Date **28-Nov-2025**

COI

Thank you for your comments and revisions. I have provided feedback about the changes made and what is still needed.

Below are the three comments that I made about the paper, along with the authors' responses. I have also then included what appears in the revised manuscript, along with my further comments:

Comment: The rigour assessment for the included studies was not detailed; inclusion was based on relevance rather than formal appraisal of methodological quality. This may result in studies of varying rigour being included, thus potentially affecting confidence in the findings. Suggest that this be considered and noted as a limitation. Response: this has been considered and noted as a limitation in the 'limitations' section of the manuscript (p13).

This synthesis integrated service user and staff perspectives to develop an evidence-based framework for understanding the mechanisms of change associated with the key relational components of peer support. This should support training, supervision, and delivery of peer support services. This was developed from the synthesis of the current evidence base. The resulting framework would benefit from direct empirical testing and refinement.

It is recommended that the statement "*The resulting framework would benefit from direct empirical testing and refinement."* does not address the question of rigour adequately. How would the researchers suggest that such empirical testing and refinement occur? While the issue of rigour is alluded to in the revised manuscript, it is important to name and explain this issue further.

Comment: Use of CMOC (Context-Mechanism-Outcome Configurations) and retroductive theorising is appropriate, but it would be good to have some information about how raw data were modelled into these theoretical configurations, and how competing explanations were considered or ruled out. Response: More information has been added to the 'coding and analysis' section on how theoretical configuration were achieve – including how competing explanations were ruled out (p6).

Thank you, what has been included in the revised manuscript addresses this comment.

Comment: While the included papers may not have included anything regarding the unintended or negative outcomes of peer support, this is an important issue when considering the effects and impact of support groups. Moreover, realist syntheses can be more complete and thus impactful when they address challenges or situations where interventions may not work as intended and/or where negative outcomes are noted and acknowledged. It would therefore be good to have this issue identified in the discussion, at least. Response: Thank you for this. A discussion of this has been added to the 'limitations' section where this has been noted as a limitation that warrants further research (p13).

I am not sure what the researchers refer to when they comment that *"A discussion of this has been added to the 'limitations' section where this has been noted as a limitation that warrants further research."* If they are referring to *"Future research could expand the transferability of the findings from the current synthesis."*, that has been included as a limitation, this is inadequate. By presenting an overly positive view of peer support in this manner is problematic. This may be the case in the included papers and their synthesis, but recognition of the dynamic and at times negative effects of support require explicit mention.

VERSION 2 - AUTHOR RESPONSE

Thank you to the reviewer who re-reviewed the article. We have made the following revisions in response to the reviewer's comments:

Comment: The rigour assessment for the included studies was not detailed; inclusion was based on relevance rather than formal appraisal of methodological quality. This may result in studies of varying rigour being included, thus potentially affecting confidence in the findings. Suggest that this be considered and noted as a limitation. Response: this has been considered and noted as a limitation in the 'limitations' section of the manuscript (p13).

This synthesis integrated service user and staff perspectives to develop an evidence-based framework for understanding the mechanisms of change associated with the key relational components of peer support. This should support training, supervision, and delivery of peer support services. This was developed from the synthesis of the current evidence base. The resulting framework would benefit from direct empirical testing and refinement.

It is recommended that the statement "The resulting framework would benefit from direct empirical testing and refinement." does not address the question of rigour adequately. How would the researchers suggest that such empirical testing and refinement occur? While the issue of rigour is alluded to in the revised manuscript, it is important to name and explain this issue further.

This is what previously added to address this comment:

In accordance with realist review methodology, inclusion of articles was based on realist assessments of relevance and rigour, rather than a formal appraisal of quality. This may have resulted in the inclusion of studies of varying rigour.

This has been expanded in the current version of the manuscript (p12 of the unmarked/clean version of the manuscript):

In accordance with realist review methodology, inclusion of articles was based on realist assessments of relevance and rigour to theory building and refinement13, rather than a formal appraisal of quality. Formal assessments of rigour using rating scales, as commonly practiced in systematic reviews, are not suitable for theory-

driven reviews. In realist analysis, which is iterative and incorporates the perspectives of people with content, lived, and professional expertise, evaluations of the trustworthiness and credibility of resulting programme theories are embedded throughout the process[13]. Although ratings scales might have classified the quality of some data as 'low', the collective insights from the included studies contributed to a conceptually robust programme theory ready for further testing.

Comment: While the included papers may not have included anything regarding the unintended or negative outcomes of peer support, this is an important issue when considering the effects and impact of support groups. Moreover, realist syntheses can be more complete and thus impactful when they address challenges or situations where interventions may not work as intended and/or where negative outcomes are noted and acknowledged. It would therefore be good to have this issue identified in the discussion, at least. Response: Thank you for this. A discussion of this has been added to the 'limitations' section where this has been noted as a limitation that warrants further research (p13).

I am not sure what the researchers refer to when they comment that "*A discussion of this has been added to the 'limitations' section where this has been noted as a limitation that warrants further research."* If they are referring to *"Future research could expand the transferability of the findings from the current synthesis."*, that has been included as a limitation, this is inadequate. By presenting an overly positive view of peer support in this manner is problematic. This may be the case in the included papers and their synthesis, but recognition of the dynamic and at times negative effects of support require explicit mention.

This is what previously added to address this comment:

This synthesis did not actively include findings or develop theory on the unintended or potential negative outcomes of peer support. This is a limitation, as this would have provided a more complete picture on when and why peer support interventions may not work as intended and/or where negative outcomes are observed. We recognise this as a gap and recommend future research to explicitly attend to potential unintended consequences or harms to inform the development of more nuanced programme theory.

This has been expanded in the current manuscript (p12 of the unmarked/clean version of the manuscript):

This synthesis did not actively incorporate findings or develop theory regarding unintended or potential negative outcomes such as those identified by a systematic review and meta-analysis of group peer support interventions[57]. This represents a limitation, as such insights could have provided a more comprehensive understanding of when and why peer support interventions may fail to achieve their intended effects or result in adverse outcomes. We acknowledge this gap and

recommend future research to explicitly examine potential unintended consequences or harms to inform the development of more nuanced programme theory.

We hope that these revisions are considered sufficient to address the concerns raised by the reviewer. Please do let us know if any additional revisions are required.
